# SIRT4 is essential for metabolic control and meiotic structure during mouse oocyte maturation

**DOI:** 10.1111/acel.12789

**Published:** 2018-05-29

**Authors:** Juan Zeng, Manxi Jiang, Xinghan Wu, Feiyang Diao, Danhong Qiu, Xiaojing Hou, Haichao Wang, Ling Li, Chunling Li, Juan Ge, Jiayin Liu, Xianghong Ou, Qiang Wang

**Affiliations:** ^1^ State Key Laboratory of Reproductive Medicine Nanjing Medical University Nanjing China; ^2^ Fertility Preservation Laboratory Human Reproduction Medical Center Guangdong Second Provincial General Hospital Guangzhou China; ^3^ Clinical Center of Reproductive Medicine First Affiliated Hospital Nanjing Medical University Nanjing China

**Keywords:** aging, meiosis, metabolism, oocyte, SIRT4

## Abstract

SIRT4 modulates energy homeostasis in multiple cell types and tissues. However, its role in meiotic oocytes remains unknown. Here, we report that mouse oocytes overexpressing SIRT4 are unable to completely progress through meiosis, showing the inadequate mitochondrial redistribution, lowered ATP content, elevated reactive oxygen species (ROS) level, with the severely disrupted spindle/chromosome organization. Moreover, we find that phosphorylation of Ser293‐PDHE1α mediates the effects of SIRT4 overexpression on metabolic activity and meiotic events in oocytes by performing functional rescue experiments. By chance, we discover the SIRT4 upregulation in oocytes from aged mice; and importantly, the maternal age‐associated deficient phenotypes in oocytes can be partly rescued through the knockdown of SIRT4. These findings reveal the critical role for SIRT4 in the control of energy metabolism and meiotic apparatus during oocyte maturation and indicate that SIRT4 is an essential factor determining oocyte quality.

## 1 INTRODUCTION

Oocyte maturation is regulated by a vast number of intra‐and extraovarian factors. The first meiotic division is initiated during embryonic development or around birth, and then the oocytes are arrested at the diplotene stage. At this stage, oocytes have a large nucleus termed as germinal vesicle (GV). Following the stimulation by luteinizing hormone (LH), chromatin condensation and disintegration of the nuclear membrane occur (germinal vesicle breakdown, GVBD). Then, the oocytes enter into metaphase I (MI) stage, at which microtubules assemble into the barrel‐shaped bipolar spindle and chromosomes align at the equatorial plate (Moor, Dai, Lee, & Fulka, 1998). After chromosome separation, oocytes extrude first polar body (PB1) and are arrested at metaphase II (MII) stage waiting for fertilization. Any error in this process can lead to the failure of meiosis so that the oocyte cannot mature, which in humans is a major cause of pregnancy loss and developmental disabilities (Hassold & Hunt, 2001).

In oocyte ooplasm, mitochondria are the richest maternal‐inherited organelle. Mitochondria are responsible for generating the majority of cellular ATP through oxidative metabolism. One well‐known side effect of oxidative phosphorylation (OXPHOS) is the generation of reactive oxygen species (ROS) that can damage diverse biological macromolecules (Wallace, Fan, & Procaccio, 2010). Thus, mitochondrial status has been recognized as an important factor determining mammalian oocyte quality. In particular, the factors associated with mitochondrial metabolism, such as ATP, ROS, and pyruvate dehydrogenase complex (PDH), are required for proper spindle assembly and chromosome alignment in oocyte meiosis (Choi et al., 2007; Johnson, Freeman, Gardner, & Hunt, 2007; Zhang, Wu, Lu, Guo, & Ma, 2006).

Sirtuins (SIRTs 1‐7) are protein deacetylases/ADP ribosyltransferases involved in various biological functions (Donmez & Guarente, 2010; Saunders & Verdin, 2009). They can be divided into nuclear (SIRT1, SIRT6, and SIRT7), mitochondrial (SIRT3, SIRT4, and SIRT5), and cytosolic (SIRT2) forms, although this division is not unconditional and some sirtuins are detected in more than one cell compartment (Michishita, Park, Burneskis, Barrett, & Horikawa, 2005). The extranuclear sirtuins in mitochondria and cytosol involve in the metabolic reactions and antioxidative defense mechanisms (Poulose & Raju, 2015). Of them, SIRT4 was shown to be critical for the maintenance of cell metabolism and DNA damage response via modifying glutamate dehydrogenase in mitochondria (Laurent et al., 2012). SIRT4 was also identified as a lipoamidase that inhibits pyruvate dehydrogenase complex (Mathias et al., 2014). However, the function of SIRT4 in mammalian oocytes remains unknown. In this study, by employing knockdown and overexpression analysis, we identified SIRT4 expression, as an essential indicator for oocyte quality, participates in the regulation metabolic function and meiotic apparatus in mouse oocytes.

## 2 RESULTS

### 2.1 Cellular localization of SIRT4 during oocyte maturation

**Figure 1:**
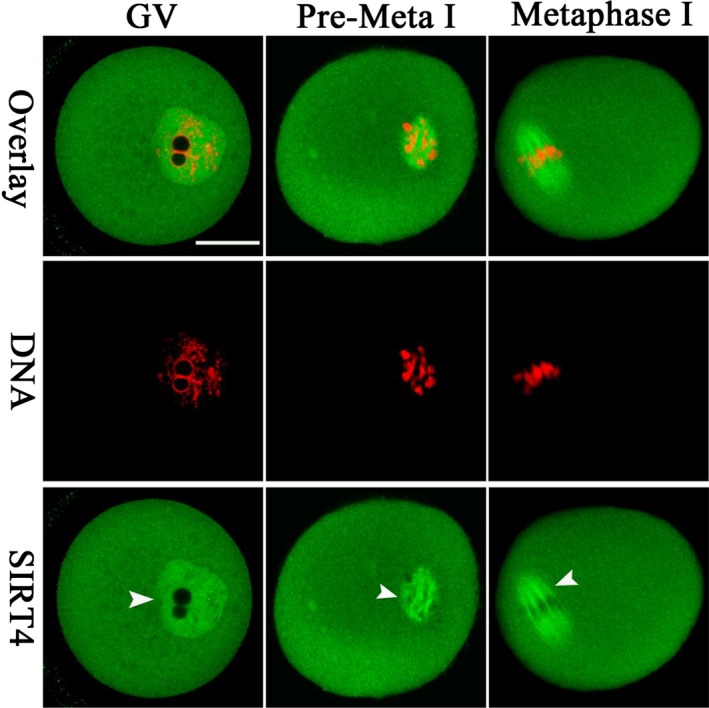
Cellular localization of SIRT4 in mouse oocytes. Oocytes at GV, Premetaphase I, and metaphase I stages were immunolabeled with SIRT4 antibody (green) and counterstained with PI to visualize DNA (red). Arrowheads point to the accumulated SIRT4 signals. Scale bar: 25 μm

We examined the cellular distribution of SIRT4 at different stages of mouse oocyte by immunostaining. As shown in Figure 1, SIRT4 resides in the entire GV oocytes, with marked accumulation in the nucleus arrowhead. As the oocytes resume meiosis, SIRT4 signals appear to be clustered around the chromosomes at premetaphase I stage. In an interesting manner, when the oocytes enter into metaphase I (MI) stage, SIRT4 primarily localizes on the spindle region and its poles (arrowhead). This dynamic distribution pattern implies that SIRT4 may participate in regulating oocyte meiotic maturation.

### 2.2 Perturbed meiotic progression in oocytes overexpressing SIRT4

**Figure 2:**
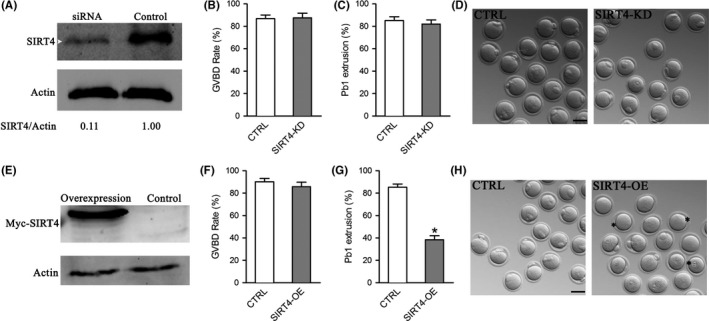
Effects of SIRT4 expression on the meiotic progression of mouse oocytes. Fully grown oocytes injected with SIRT4‐cRNAs or SIRT4‐siRNAs were arrested in medium with milrinone for 20 hr, washed in milrinone‐free medium, and then cultured in vitro for the following analysis. (A) Efficiency of SIRT4 knockdown (SIRT4‐KD) after siRNA injection was verified by immunoblotting. Actin served as a loading control. Band intensity was calculated using ImageJ software, and the ratio of SIRT4/Actin expression was normalized and values are indicated. (B–C) Quantitative analysis of GVBD and Pb1 extrusion rate in control (*n* = 137) and SIRT4‐KD (*n* = 112) oocytes. (D) Representative images of control and SIRT4‐KD oocytes. (E) Efficiency of SIRT4 overexpression (SIRT4‐OE) after cRNA injection was verified by immunoblotting. (F–G) Quantitative analysis of GVBD and Pb1 extrusion rate in control (*n* = 120) and SIRT4‐OE (*n* = 122) oocytes. (H) Representative images of control and SIRT4‐OE oocytes. Asterisks indicate the oocytes that fail to extrude polar body. The graph shows the mean ± *SD* of the results obtained in three independent experiments. *p <* *0.05 vs. controls. Scale bar: 100 μm

**Figure 3:**
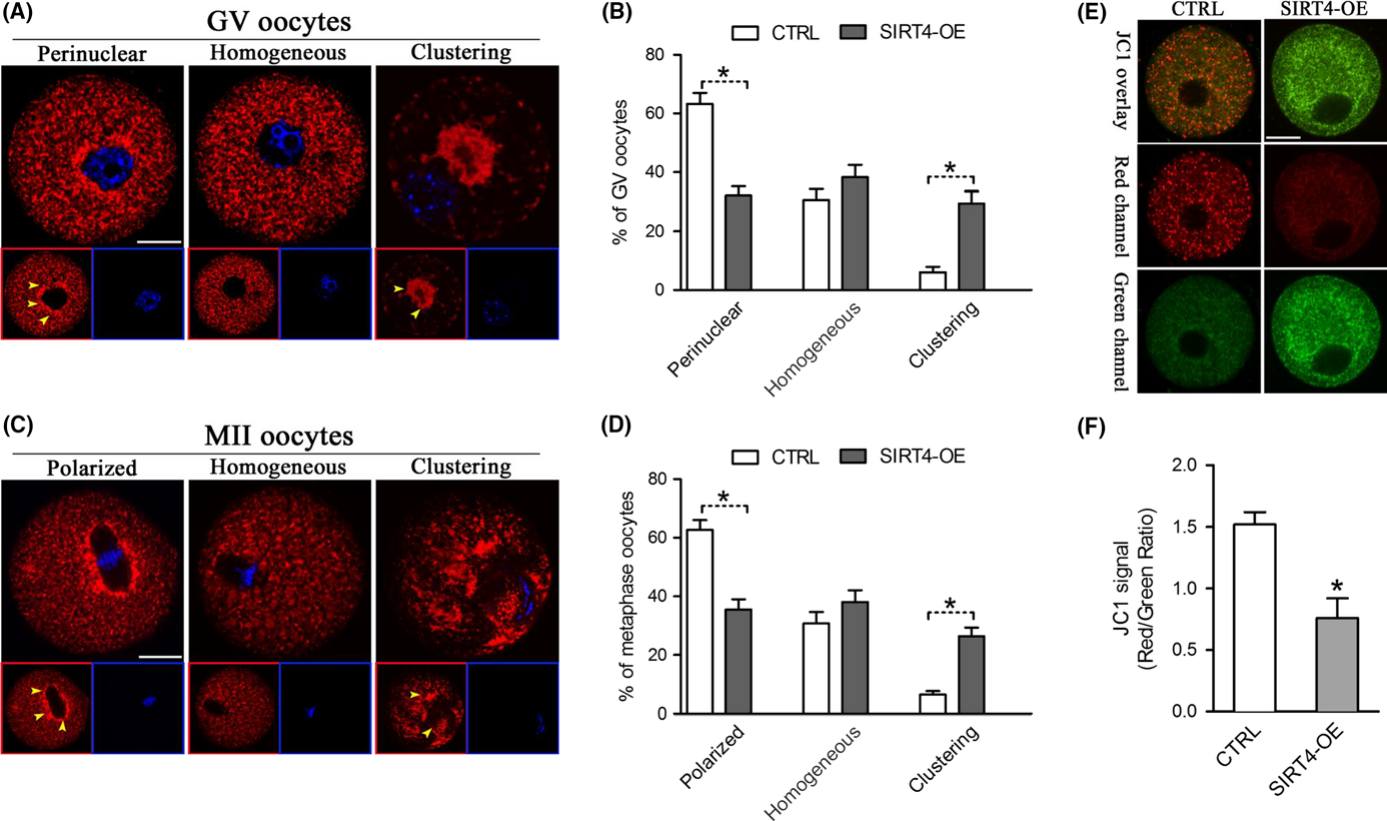
SIRT4 overexpression disrupts mitochondrial redistribution and membrane potential in oocyte. Control and SIRT4‐OE oocytes were labeled with MitoTracker Red to visualize mitochondrial localization and counterstained with Hoechst 33342 to show meiotic stages. Mitochondrial distribution patterns were evaluated using confocal microscopy. (A) Representative images of mitochondrial distribution patterns in GV oocytes: perinuclear distribution, homogeneous distribution, and clustering distribution. (B) Quantification of control and SIRT4‐OE oocytes at GV stage with each mitochondrial distribution pattern. (C) Representative images of mitochondrial distribution patterns in MII oocytes: polarized distribution, homogeneous distribution, and clustering distribution. (D) Quantification of control and SIRT4‐OE oocytes at MII stage with each mitochondrial distribution pattern. (E) Mitochondrial membrane potential in control and SIRT4‐OE oocytes measured by JC‐1 fluorescence. The green fluorescence shows inactive mitochondria and the red fluorescence shows active mitochondria in oocytes. (F) Histogram showing the JC‐1 red/green fluorescence ratio. Error bars indicate ± *SD*. *p <* *0.05 vs. control. Scale bars: 25 μm

To explore the roles of SIRT4 in meiosis, fully grown immature oocytes were microinjected with specifically designed siRNAs to knock down the SIRT4 level (SIRT4‐KD). Immunoblotting showed that siRNA injection led to a significant decrease in SIRT4 protein expression in oocytes (Figure 2A). The efficiency of SIRT4 knockdown was also confirmed by quantitative RT‐PCR and immunostaining (Supporting Information Figure S2). Nonetheless, we noted that neither meiotic resumption nor PB1 emission was affected in SIRT4‐KD oocytes, as shown in Figure 2B–D, indicating that SIRT4 depletion is dispensable for oocyte maturation. Then, we asked whether elevating SIRT4 expression influences oocyte development. To address this question, SIRT4 cRNAs were injected into oocytes and meiotic progression was analyzed. Immunoblotting verified that exogenous SIRT4 protein was efficiently overexpressed (Figure 3E). As shown in Figure 2F–H, SIRT4 overexpression (SIRT4‐OE) did not affect meiotic resumption significantly, but reduced the percentage of PB1 extrusion in oocytes compared to controls. To ensure the effects observed are specific to SIRT4, we constructed the dominant negative mutant of SIRT4 (SIRT4‐H158Y) and then injected into mouse oocytes. As shown in Supporting Information Figure S1A–C, no apparent effects on meiotic progression and morphology were detected in these oocytes. Meanwhile, we also performed a rescue experiment and found that SIRT4‐siRNA injection could efficiently prevent the maturational defects of SIRT4‐cRNA‐injected oocytes (Supporting Information Figure S1D). These results suggest that ectopic expression of SIRT4 perturbs the meiotic progress in mouse oocytes.

### 2.3 SIRT4 overexpression influences mitochondrial redistribution and membrane potential

Mitochondrial dynamics have been indicated to be necessary for producing a competent oocyte. We next determined whether mitochondrial localization was altered during SIRT4‐OE oocyte maturation. GV and MII stage oocytes were labeled with MitoTracker, and mitochondrial dynamics were evaluated by confocal microscopy. As we described previously (Wang et al., 2009), there are mainly three different patterns of mitochondrial distribution in GV and MII oocytes (Figure 3A,C), including perinuclear (GV)/polarized (MII) distribution, homogeneous distribution, and clustering distribution. The majority of normal immature oocytes present perinuclear distribution pattern, characterized by the apparent accumulation of mitochondria around GV. As the meiotic maturation is completed, mitochondria display a polarized distribution pattern in most MII oocytes, characterized by the enriched localization around the spindle. It is remarkable that, we found that the proportion of oocytes with clustering mitochondria was significantly increased when SIRT4 was overexpressed, whereas the proportion of perinuclear/polarized distribution pattern was reduced accordingly in comparison with controls (Figure 3B,D). These results suggest that elevated SIRT4 expression leads to the inadequate redistribution of mitochondria during oocyte maturation.

Meantime, we analyzed the mitochondrial status using the fluorescent dye JC‐1, which shifts from green to red with increasing membrane potential (Δψ_m_) (Wang et al., 2010). Mitochondria in control oocytes generally had predominantly high Δψ_m_ as indicated by the red fluorescence, while a trend toward lower Δψ_m_ was observed in SIRT4‐OE oocytes (Figure 3E). Quantitative analysis further confirmed that the red/green ratio was significantly decreased in SIRT4‐overexpressing oocytes as compared to their controls (Figure 3F). Altogether, the results suggest that ectopic expression of SIRT4 disrupts mitochondrial dynamics and membrane potential.

### 2.4 SIRT4 overexpression leads to metabolic dysfunction in oocytes

**Figure 4:**
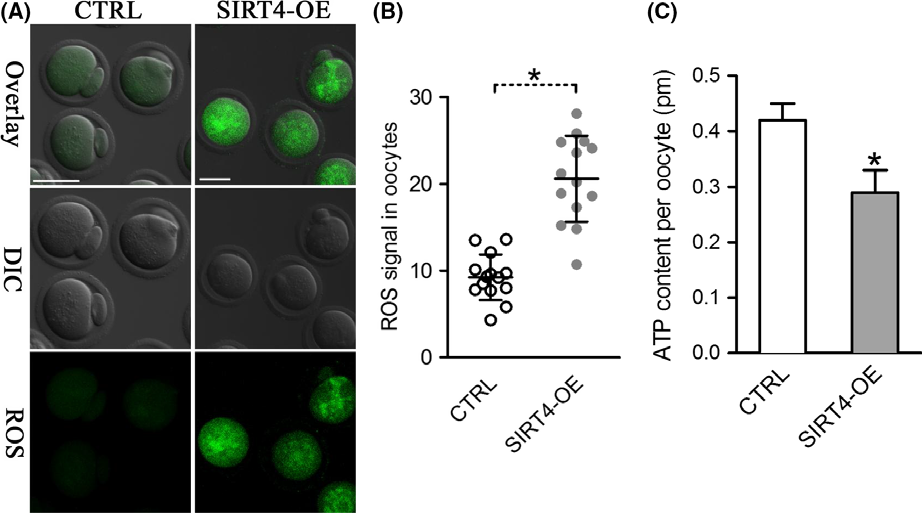
SIRT4 overexpression causes metabolic dysfunction in oocytes. (A) Representative images of CM‐H2DCFAD fluorescence in control and SIRT4‐OE oocytes. Scale bar: 50 μm. (B) Quantitative analysis of fluorescence intensity shown in panel A (*n* = 14 for each group). (C) Histogram showing the ATP level in control and SIRT4‐OE oocytes (*n* = 20 for each of the three trials). Data are expressed as the mean ± *SD*, student *t* test was performed on the three means of the experiments. *p <* *0.05 vs. controls

Having shown that SIRT4 overexpression disrupts the mitochondrial redistribution, we decided to check whether mitochondrial function is influenced accordingly. Mitochondria are the major reactive oxygen species (ROS) producer, as well as one of the main targets of ROS‐induced oxidative damage (Ramalhosantos et al., 2009). Therefore, we first assayed the ROS content in live oocytes using CM‐H2DCFDA fluorescent dye. As shown in Figure 4A,B, the ROS signals were markedly elevated in SIRT4‐OE oocytes relative to control cells. Furthermore, we measured the ATP levels in control and SIRT4‐OE oocytes by conducting microanalytical assays. Results showed that SIRT4 overexpression led to a ~30% reduction in ATP content compared to controls (Figure 4C). These data suggest that SIRT4 is an essential factor modulating oocyte metabolism.

### 2.5 SIRT4 overexpression induces meiotic defects during oocyte maturation

**Figure 5:**
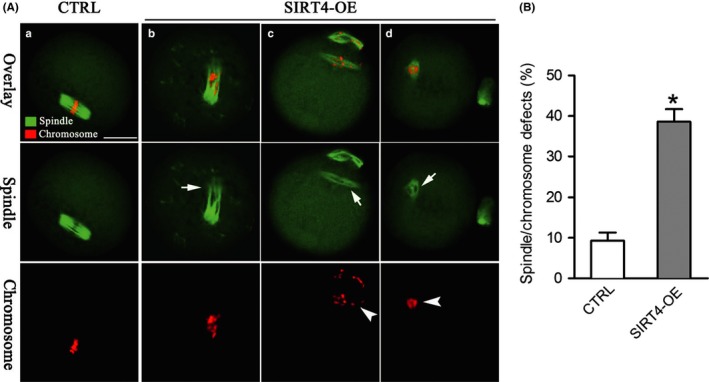
SIRT4 overexpression induces spindle defects and chromosome misalignment in oocyte meiosis. (A) Control (a) and SIRT4‐OE (b–d) oocytes were stained with α‐tubulin antibody to visualize the spindle (green) and counterstained with PI (red) to observe chromosomes. Arrows indicate spindle defects and arrowheads indicate chromosome misalignment in MII oocytes. (B) Quantification of control and SIRT4‐OE oocytes with spindle/chromosome defects. Data are expressed as mean percentage ± *SD* from three independent experiments in which at least 120 oocytes were analyzed. *p <* *0.05 vs. controls. Scale bars: 20 μm

The specific localization of SIRT4 during oocyte maturation prompted us to ask whether SIRT4 functions in the assembly of meiotic structure. To address this question, control and SIRT4‐OE oocytes were labeled with antitubulin antibody to visualize the spindle and costained with PI for chromosomes. Most control metaphase oocytes presented a typical barrel‐shaped spindle and well‐aligned chromosomes at the equatorial plate (Figure 5Aa). By contrast, SIRT4‐OE oocytes frequently showed chromosome misalignment (arrowheads) and spindle disorganization (arrows) (Figure 5Ab,c,d). Quantitative analysis demonstrated that the proportion of SIRT4‐OE oocytes with spindle/chromosome defects was significantly higher than that of control cells (Figure 5B). Together, these findings suggest that SIRT4 plays an important role in microtubule stability and chromosome organization during mouse oocyte maturation.

### 2.6 Phosphorylation of Ser293‐PDHE1α mediates the effects of SIRT4 overexpression on oocyte metabolism

**Figure 6:**
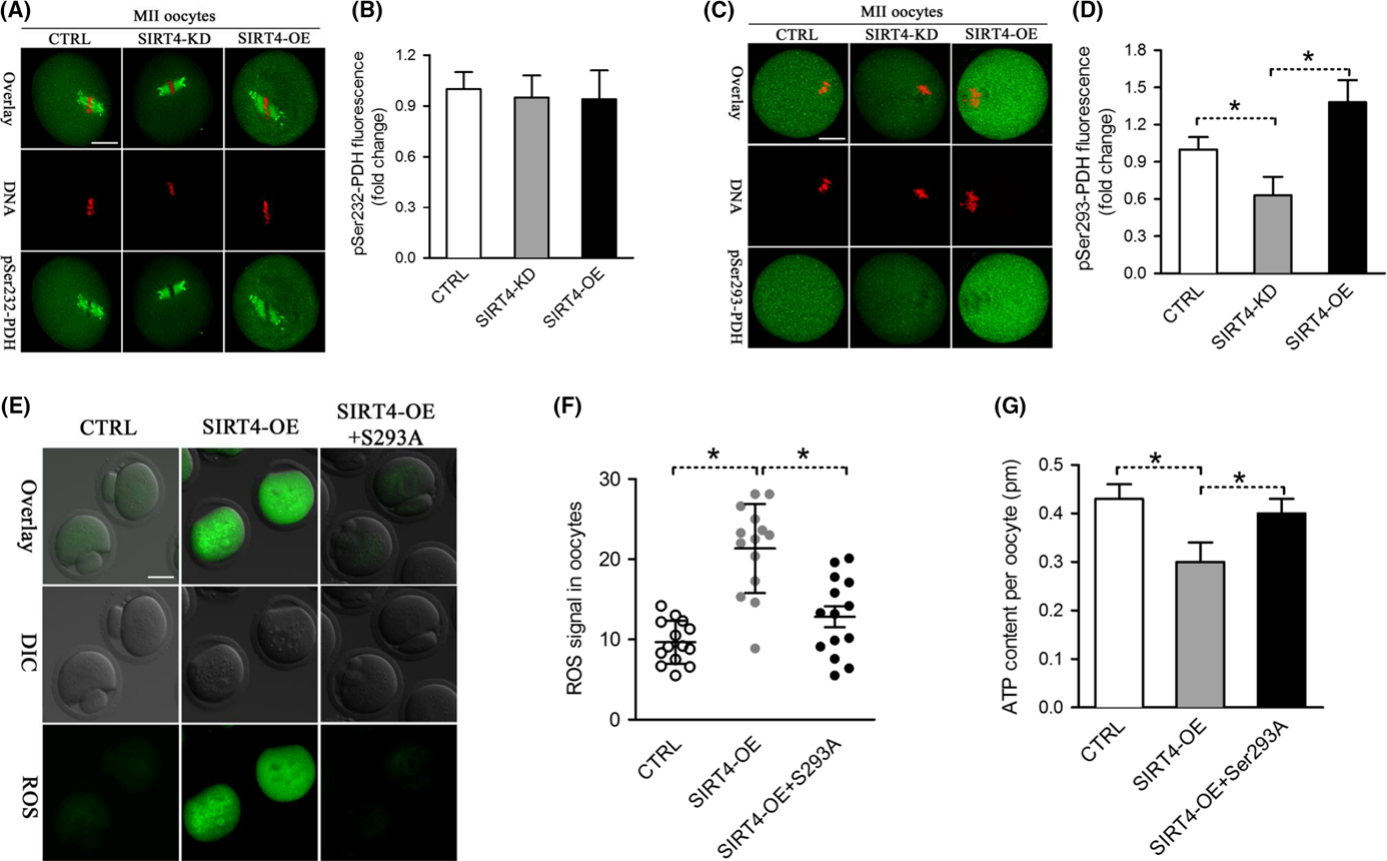
Phosphorylation of Ser293‐PDHE1α mediates the effects of SIRT4 on oocyte metabolism. (A) Control, SIRT4‐KD, and SIRT4‐OE oocytes were stained with pSer232‐PDHE1α antibody (green) and costained with PI (red) for DNA. (B) Quantification of pSer232‐PDHE1α fluorescence in (A). (C) Control, SIRT4‐KD, and SIRT4‐OE oocytes were labeled with pSer293‐PDHE1α antibody (green) and costained with PI (red) for DNA. (D) Quantification of pSer293‐PDHE1α fluorescence in (C). (E) Representative images of CM‐H2DCFDA fluorescence (green) in control, SIRT4‐OE, and SIRT4‐OE+Ser293A mutant oocytes. (F) Quantification of the relative level of ROS in oocytes. Each data point represents an oocyte (*n* = 14 for each group). (G) Histogram showing the ATP level in control, SIRT4‐OE, and SIRT4‐OE+Ser293A mutant oocytes (*n* = 20 for each of the three trials). Data are expressed as the mean ± *SD*, student *t* test was performed on the three means of the experiments. *p <* *0.05 vs. controls. Scale bars: 25 μm

Of note, ectopic expression of SIRT4 results in the very similar phenotypes as we observed in oocytes overexpressing pyruvate dehydrogenase kinase 3 (PDK3) (Hou et al., 2015). Moreover, it has been reported that PDKs expression was altered in multiple cell types with SIRT4 depletion (Nasrin et al., 2010; Tao, Zhang, Ling, McCall, & Liu, 2018). PDKs modulate energy homeostasis in diverse tissues through phosphorylation of the pyruvate dehydrogenase (PDH) complex. In an interesting manner, SIRT4 has been identified as a guardian of cellular metabolism by regulating PDH activity (Mathias et al., 2014). PDH is able to modulate tricarboxylic acid (TCA) cycle in cells, producing ATP and many biosynthetic intermediates in mammalian oocytes. We have discovered that the phosphorylation status of PDHE1α subunit is associated with metabolic control and meiotic apparatus in mouse oocytes (Hou et al., 2015). We therefore asked whether the PDHE1α phosphorylation, at least in part, mediates the effects of SIRT4 on oocyte maturation. Toward this goal, we first examined the phosphorylation status of two serine residues (Ser232 and Ser293) on PDHE1α in control and SIRT4‐OE oocytes. pSer232‐PDHE1α accumulated on the spindle region and its poles of metaphase oocytes (Figure 6A,B) as we reported previously (Hou et al., 2015). However, neither SIRT4 knockdown nor overexpression showed an apparent effect on the pSer232‐PDHE1α signal intensity. In marked contrast, we found that ectopic expression of SIRT4 enhanced pSer293‐PDHE1α signal in the cytoplasm, and pSer293‐PDHE1α staining was significantly decreased in SIRT4‐KD oocytes (Figure 6C,D).

We next performed a functional rescue experiment to test whether SIRT4 overexpression‐induced oxidative stress is through the phosphorylation of Ser293‐PDHE1α. Serine 293 on PDHE1α was mutated to an alanine residue (S293A) to preclude phosphorylation. SIRT4 cRNAs and S293A mutant cRNAs were simultaneously injected into oocytes and metabolic phenotypes were evaluated. As shown in Figure 6E,F, the elevated ROS levels in SIRT4‐OE oocytes were partly prevented by the co‐expression of nonphosphorylatable S293A mutant. Similar to that, the ATP content in SIRT4‐OE oocytes was almost restored to normal levels (Figure 6G). Due to the limitation of oocyte number, we have not yet been able to directly dissect the relationship between PDHE1α phosphorylation and SIRT4 activity in mouse oocytes. Together, these findings indicate that phosphorylation of Ser293‐PDHE1α is important for SIRT4‐controlled oocyte metabolism.

### 2.7 Reducing SIRT4 expression alleviates the deficient phenotypes of oocytes from aged mice

**Figure 7:**
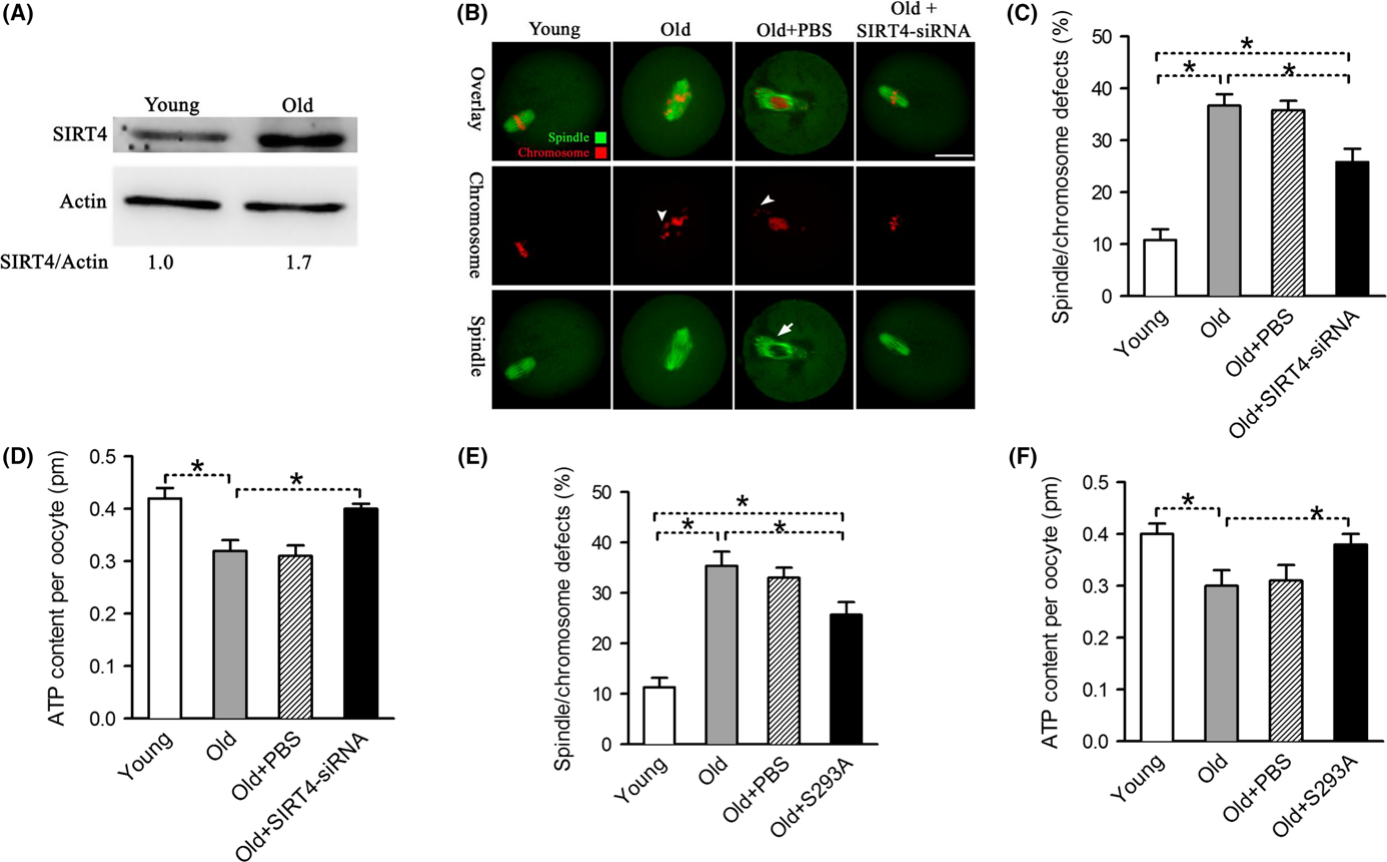
SIRT4 upregulation contributes to the maternal age‐associated defective phenotypes in oocytes. (A) Immunoblotting analysis shows the increased SIRT4 expression in ovulated MII oocytes from aged females compared to young controls. Actin served as a loading control. Band intensity was calculated using ImageJ software, and the ratio of SIRT4/Actin expression was normalized and values are indicated. (B) Representative examples of meiotic spindle and chromosomes at MII stage in young, old, old+PBS, and old+SIRT4‐siRNA oocytes. Arrowheads indicate the misaligned chromosomes and arrow indicates the defect spindles. Scale bars: 25 μm. (C) Quantification of young, old, old+PBS, and old+SIRT4‐siRNA oocytes with spindle/chromosome defects. Data are expressed as mean percentage ± *SD* from three independent experiments in which at least 120 oocytes were analyzed. (D) Histogram showing the ATP level in young, old, old+PBS, and old+SIRT4‐siRNA oocytes (*n* = 20 for each of the three trials). (E) Quantification of young, old, old+PBS, and old+S293A oocytes with spindle/chromosome defects. Data are expressed as mean percentage ± *SD* from three independent experiments in which at least 120 oocytes were analyzed. (F) Histogram showing the ATP level in young, old, old+PBS, and old+S293A oocytes (*n* = 20 for each of the three trials). Data are expressed as the mean ± *SD*, student *t* test was performed on the three means of the experiments. *p <* *0.05 vs. controls

Female fertility decreases with advanced maternal age largely due to the meiotic abnormality in oocyte. Here, we noticed that SIRT4‐OE oocytes showed very similar phenotypes as aged oocytes. To test whether maternal age affects the SIRT4 expression in oocytes, we established a natural aging mouse model (42–45 weeks). Oocytes from young (6–8 weeks) and old mice were collected for western blotting analysis. In a surprising way, we found that SIRT4 level increases in old oocytes compared to their young controls (Figure 7A). We next conducted knockdown experiments to investigate whether reducing SIRT4 expression in old oocytes could partly rescue their deficient phenotypes. As shown in Figure 7B,C, the frequency of spindle/chromosome defects was significantly higher in old oocytes than that in young oocytes. It is note that, these abnormalities were decreased when SIRT4 was knocked down in old oocytes. Similar that, lowered SIRT4 expression was able to restore the ATP generation to approximately normal levels, as shown in (Figure 7D). In addition, consistent with the data in Figure 6, we found that nonphosphorylatable S293A mutant also could partly rescue the phenotypes of old oocytes (Figure 7E,F). Taken together, these results suggest that the elevated SIRT4 expression contributes to the metabolic dysfunction and meiotic defects in oocytes from aged mice.

## 3 DISCUSSION

SIRT4 is located primarily in the mitochondrial matrix and involved in a variety of events in mitotic cells (Fu et al., 2016). Differential roles of SIRT4 have been reported in diverse cell types and multiple tissues. Here, we found that SIRT4 presents a meiosis‐specific distribution in mouse oocytes (Figure 1). In an interesting manner, SIRT4 overexpression, not knockdown, induces inadequate mitochondrial redistribution and over‐production of ROS in oocytes (Figures 2, 3, 4). Similar to this, ectopic expression of SIRT4 has also been reported to negatively influence mitochondrial quality control in other tissues (Castex et al., 2017; de Moura, Uppala, Zhang, Van Houten, & Goetzman, 2014; Luo et al., 2016). In mammalian oocytes and embryos, mitochondria are the most prominent organelles, which house the respiratory chain for the generation of cellular energy and are the site of essential biosynthetic pathways. During oocyte maturation, there are marked changes in the distribution of mitochondria that supply the majority of the cellular ATP (Yu, Dumollard, Rossbach, Lai, & Swann, 2010). The difference in mitochondria localization has been associated with developmental competence of oocytes (Ferreira et al., 2009). The mitochondrial membrane potential must be maintained to support ATP synthesis; however, a high potential will favor the production of deleterious ROS (Merry, 2002). It is worth noting that mitochondrial function and redox state are important for proper assembly of meiotic structure during oocyte meiosis. Chromatin condensation during meiosis is an ATP‐dependent process. Germinal vesicle breakdown and formation of metaphase spindles also may have higher energy demands. Emerging evidence has suggested that insufficient ATP availability and increased oxidative stress are tied to spindle disorganization and chromosome misalignment in oocytes (Eichenlaub‐Ritter, Vogt, Yin, & Gosden, 2004; Tilly & Sinclair, 2013; Zeng et al., 2007). In line with this conception, we observed the high frequency of meiotic defects in oocytes overexpressing SIRT4 (Figure 5). Therefore, we propose that SIRT4 is an essential meiotic regulator through the control of mitochondrial function.

Throughout the growth period, pyruvate and oxygen consumption is gradually increased in mouse oocytes (Harris, Leese, Gosden, & Picton, 2009). PDH is able to convert pyruvate into acetyl coenzyme A and thereupon modulates the entry of glucose‐derived carbons into TCA cycle (Hou et al., 2015). Pdha1‐deficient mouse oocytes experience inadequate ATP levels along with chromatin and microtubular abnormalities (Johnson et al., 2007). We have demonstrated that phosphorylation of Ser293‐PDHE1α results in disruption of meiotic spindle morphology and chromosome movement and lowered total ATP content, probably through inhibition of PDH activity (Hou et al., 2015). It is note that, SIRT4 was recently found to decrease the activity of the PDH complex in human cell lines and mouse models (Mathias et al., 2014). Herein, by employing knockdown and overexpression analysis, we showed that phosphorylation status of Ser293‐PDHE1α is directly or indirectly controlled by SIRT4 expression in mouse oocytes. It is important that, Ser293A mutant could partly rescue the metabolic phenotypes in SIRT4‐OE oocytes (Figure 6). Taking together, it is tempting to conclude that SIRT4 controls redox homeostasis and meiotic apparatus in oocytes possibly by affecting the phosphorylation of Ser293‐PDHE1α. However, our data do not support the conclusion that PDH is a direct target of SIRT4 in mouse oocytes. SIRT4 may modulate the PDH‐Ser293 phosphorylation by altering PDKs expression. Determining how SIRT4 interacts with PDK or PDH would be a follow‐up project. In addition, we cannot rule out that SIRT4 may act on other distinct substrates to function during oocyte maturation.

A hallmark of animal development is an age‐related decrease in fertility. This is largely attributed to females producing eggs of compromised developmental competence, particularly with spindle/chromosome anomalies (Luke et al., 2012; Navot et al., 1994). Although multiple molecules have been identified to contribute to the maternal age effect, additional factors remain to be explored for the clinical management of fertility issues. In this study, we discovered that SIRT4 protein levels are increased in oocytes from old mouse (Figure 7). Likewise, SIRT4 expression is upregulated during senescence triggered by different stimuli in human skin cells and trophoblast stem cells (Castex et al., 2017; Lang et al., 2016). SIRT4 upregulation has also been implicated to adversely impact mitochondrial functions and contribute to the development of senescent phenotypes (Castex et al., 2017). It is important that, reducing SIRT4 or overexpressing Ser293A‐PDHE1α could ameliorate the metabolic and meiotic defects in aged oocytes (Figure 7). Accurate control of the assembly of meiotic apparatus and well‐balanced energy metabolism are the critical determinants for oocyte quality. Hence, our study indicates a potential target mediating the effects of advanced maternal age on oocyte/embryo developmental competence.

## 4 MATERIALS AND METHODS

All chemicals and culture media were purchased from Sigma (St. Louis, MO, USA) unless stated otherwise.

### 4.1 Mice

All animal experimental protocols were performed in accordance with relevant ethical guidelines and regulations, and approved by the Animal Care and Use Committee of Nanjing Medical University. ICR female mice (6–8 weeks old) were used in our experiments. To generate a natural aging model, 42‐ to 45‐week‐old female mice (near the end of their reproductive lifespan) were selected.

### 4.2 Antibodies

Rabbit polyclonal anti‐SIRT4 antibodies (Cat#: S0948), mouse monoclonal FITC‐conjugated anti‐α‐tubulin antibodies (Cat#: F2168), and mouse monoclonal anti‐β‐actin antibodies (Cat#: A5441) were purchased from Sigma; mouse monoclonal anti‐Myc antibodies (Cat#: ab18185) were purchased from Abcam; Rabbit polyclonal anti‐PDHE1α (pSerine232) antibodies and Rabbit polyclonal anti‐PDHE1α (pSerine293) antibodies were purchased from EMD Chemicals (Cat#: AP1063 and AP1062).

### 4.3 Oocyte collection and culture

Oocytes were isolated from female mice at the age of 3–4 weeks (young mice) and 42–45 weeks (reproductively old mice). To retrieve fully grown GV oocytes, mice were injected intraperitoneally with five IU Pregnant Mares Serum Gonadotropin (PMSG) for ovarian follicle stimulation. About 46–48 hr later, cumulus‐enclosed oocytes were collected by manual rupturing of antral ovarian follicles. With gentle washes by repeatedly pipetting, cumulus cells were removed and denuded oocytes were obtained. For in vitro maturation, denuded GV oocytes were cultured for 14 hr in M16 medium covered with mineral oil at 37°C in a 5% CO2 incubator.

### 4.4 Plasmid construction and mRNA synthesis

Total RNA was extracted from 100 mouse oocytes using Arcturus PicoPure RNA Isolation Kit (Applied Biosystems, CA, USA), and the cDNA was generated with QIAquick PCR Purification Kit (Qiagen, Germany). PCR products were purified, digested with *FseI* and *AscI* (NEB Inc, MA, USA), and then cloned into the pCS2^+^ vector with six Myc tags. The pCS2^+^ vectors encoding the PDHE1α and SIRT4H158Y mutants were generated with the use of a QuickChange site‐directed mutagenesis kit (Stratagene). For the synthesis of cRNA, the plasmids were linearized by *NotI*. Capped cRNAs were made using in vitro transcription with SP6 mMESSAGE mMACHINE (Ambion, CA, USA) according to the manufacturer's instruction. Synthesized RNA was aliquoted and stored at −80°C. The related primer sequences can be found in Supporting Information Table S1.

### 4.5 Knockdown and overexpression analysis

Microinjections of siRNA or mRNA were used to knock down or overexpress proteins in mouse oocytes, respectively. In knockdown experiments, SIRT4‐targeting siRNA was diluted with water to give a stock concentration of 1 mm, and 2.5 pl solution was injected. About 10 pl cRNA solution (10 ng/μl) was injected into cytoplasm of oocytes in overexpression experiments, and the same amount of RNase‐free PBS was injected as control. Following injections, oocytes were arrested at GV stage in medium containing 2.5 μM milrinone for 20 hr to promote mRNA degradation or translation. The siRNA pairs used were listed in Supporting Information Table S1.

### 4.6 Immunoblotting

A total of 150 oocytes were lysed in Laemmli buffer containing protease inhibitor. Samples were subjected to a 10% SDS–PAGE and then transferred to a polyvinyl fluoride (PVDF) membrane. Nonspecific binding sites were blocked with 5% nonfat dry milk in Tris‐buffered saline containing 0.05% Tween‐20 (TBST) for 2 hr and then probed with primary antibodies overnight at 4°C (anti‐SIRT4 antibody, 1:300; anti‐Myc antibody, 1:1,000; anti‐β‐actin antibody, 1:5,000). After three washes in TBST and incubation with HRP‐conjugated secondary antibodies, the protein bands were visualized using an ECL Plus Western Blotting Detection System (GE Healthcare, Little Chalfont, UK).

### 4.7 Immunofluorescence

Immunofluorescence was performed as described previously (Ma et al., 2014). In brief, oocytes were fixed with 4% paraformaldehyde for 30 min, permeabilized in 0.5% Triton‐X 100 for 20 min, and treated with blocking buffer (1% BSA‐supplemented PBS) for 1 hr. Samples were subjected to indirect immunofluorescence staining by incubating overnight at 4°C with primary antibodies as follows: anti‐SIRT4 antibody, anti‐PDHE1α (pSerine232) antibody, and anti‐PDHE1α (pSerine293) antibody. To visualize spindle, oocytes were probed with FITC‐conjugated tubulin antibody. For mitochondria staining, oocytes were cultured in M16 medium containing 200 nM MitoTracker Red (Molecular Probes, Eugene, OR) for 30 min at 37°C. To evaluate mitochondrial membrane potential, oocytes were cultured in M16 medium containing 2 μM JC‐1 for 30 min at 37°C. Chromosomes were counterstained with propidium iodide (PI) or Hoechst 33342 for 10 min. After three washes, samples were mounted on antifade medium (Vectashield, Vector Laboratories, CA, USA) and then examined under a laser scanning confocal microscope (LSM 710; Carl Zeiss, Germany). For each antibody used, immunofluorescence was always performed and processed on oocytes from control and treatment group in parallel, and under identical conditions. Images were always acquired using the same confocal microscope settings, even between experiments. ImageJ software (U.S. National Institutes of Health, USA) was used to quantify the intensity of fluorescence.

### 4.8 Measurement of ATP content

ATP determination was performed using a Bioluminescent Somatic Cell Assay Kit (Sigma, St. Louis, MO, USA) as we described previously (Hou et al., 2015). In brief, 20 oocytes were pooled together and processed according to the procedure described by Combelles and Albertini(Combelles & Albertini, 2003). A six‐point standard curve (0, 1.0, 2.5, 5.0, 7.5, and 10 pmol of ATP) was generated in each assay, and ATP levels were calculated using the formula derived from the linear regression of the standard curve.

### 4.9 Measurement of ROS levels

Intracellular ROS levels were determined by CM‐H2DCFDA (Cat#: C6827; Life Technologies, Invitrogen TM). CM‐H2DCFDA was prepared in DMSO prior to loading. Oocytes were incubated in M16 medium containing 5 mM CM‐H2DCFDA for 20 min at 37°C in a 5% CO_2_ incubator. Following three washes, 20 oocytes were loaded on a slide with a microdrop of medium, and immediately observed under a Laser Scanning Confocal Microscope (LSM 710, Zeiss, Germany).

### 4.10 Statistical analysis

All experiments were replicated more than three times, and the data obtained were subjected to statistical analysis. Data are presented as mean ± *SD*, unless otherwise indicated. Differences between two groups were analyzed by Student's *t* test. Multiple comparisons between more than two groups were analyzed by one‐way ANOVA test using Prism 5.0. *p *<* *0.05 was considered to be significant.

## AUTHOR'S CONTRIBUTION

JZ and QW designed research; JZ, MJ, XW, FD, DQ, XH, HW, LL, CL, and JG performed research; JZ, JL, XO, and QW analyzed data; JZ and QW wrote manuscript.

## CONFLICT OF INTEREST

None declared.
