# Supplementary material for: SIRT4 is essential for metabolic control and meiotic structure during mouse oocyte maturation
Source: Aging Cell. 2018 May 29;17(4):e12789. doi: 10.1111/acel.12789 (PMC6052465; doi:10.1111/acel.12789)
Supplement: Supplementary file 1 [file ACEL-17-na-s001.pdf]

Supplemental Figure 1

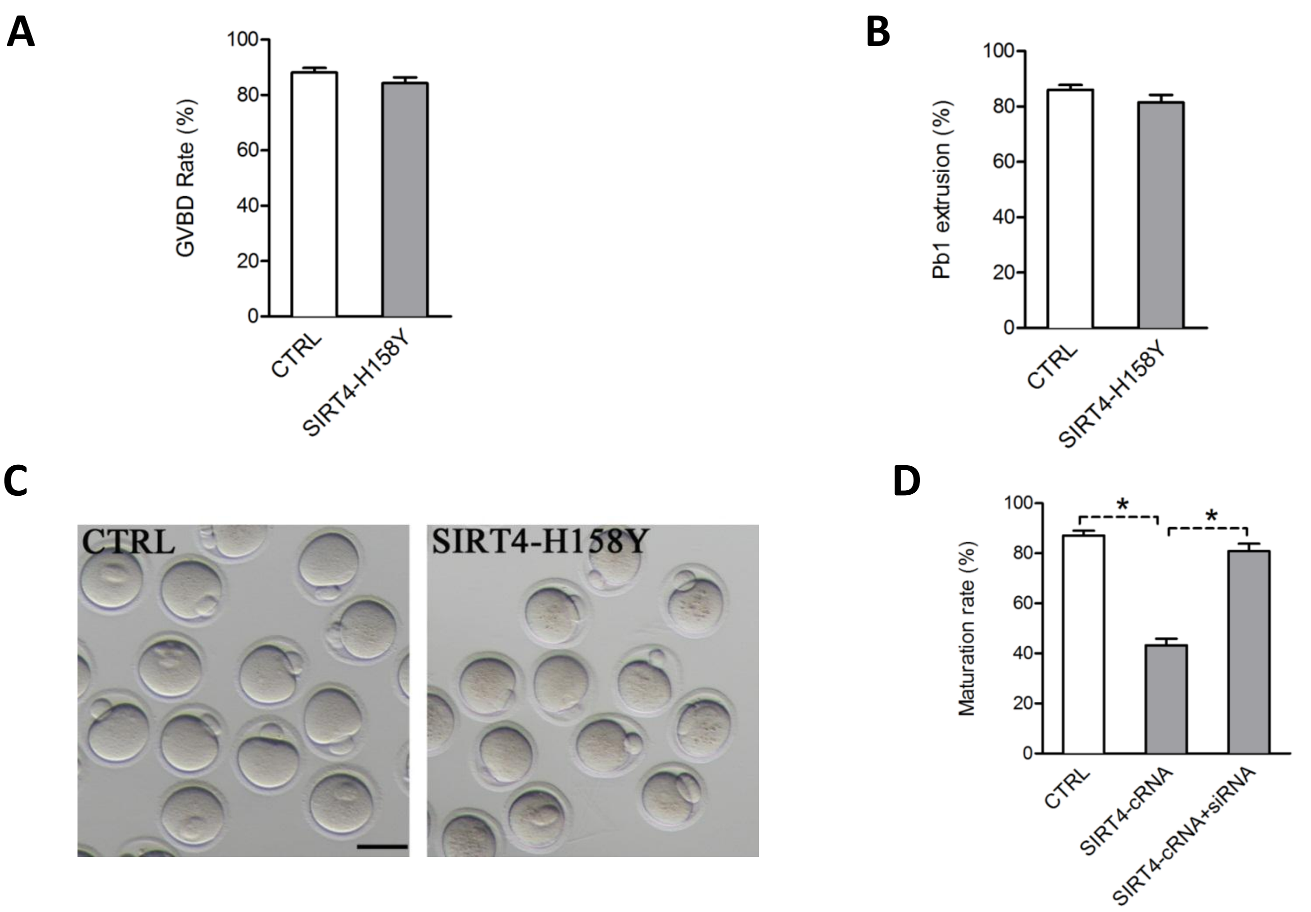

Figure S1 Effects of SIRT4-H158Y mutant on oocyte maturation. (A-B) Quantitative analysis of GVBD and Pb1 extrusion rate in control (n=102) and SIRT4-H158Y (n=106) oocytes. (C) Representative images of control and SIRT4-H158Y oocytes. Scale bar, 80 μm. (D) (B) Quantitative analysis of maturation rate in control (n=85), SIRT4-cRNA (n=80), and SIRT4-cRNA+siRNA oocytes (n=80). Results are mean ± SD. *, $P < 0.05$.
