# Supplementary material for: SIRT4 is essential for metabolic control and meiotic structure during mouse oocyte maturation
Source: Aging Cell. 2018 May 29;17(4):e12789. doi: 10.1111/acel.12789 (PMC6052465; doi:10.1111/acel.12789)
Supplement: Supplementary file 2 [file ACEL-17-na-s002.pdf]

Supplemental Figure 2

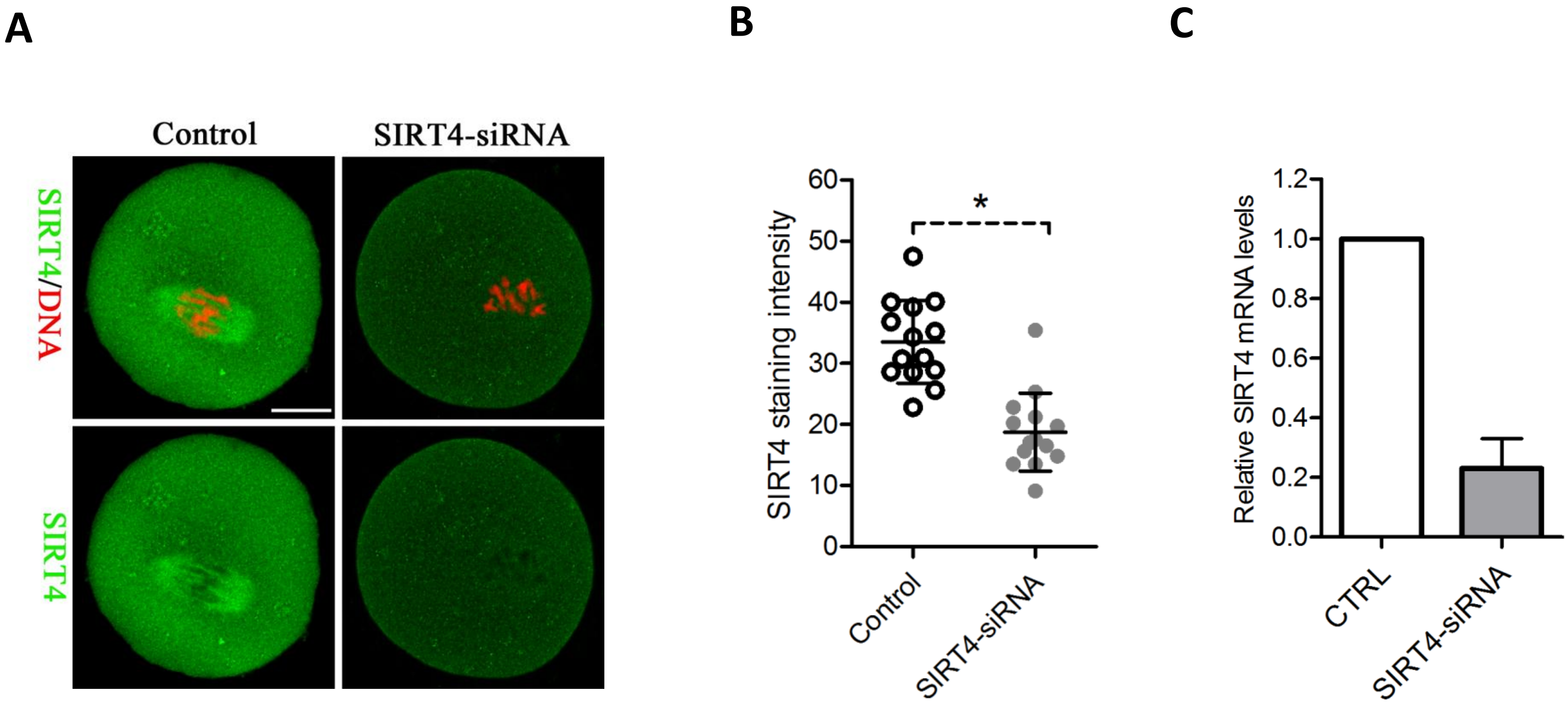

Figure S2 Effects of siRNA knockdown on SIRT4 staining in oocytes. (A) Representative confocal sections of control and SIRT4-siRNA injected oocytes stained with anti-SIRT4 antibody (green) and counterstained with propidium iodide (red) for chromosomes are shown. Scale bar, 25 µm. (B) Quantification of SIRT4 immunofluorescence shown in A (n = 15 for control and n = 15 for SIRT4-siRNA group). (C) The relative mRNA levels in control and SIRT4-siRNA injected oocytes. Experiments were repeated three times and results are mean ± SD. *, $P < 0.05$.
