# Supplementary material for: SIRT4 is essential for metabolic control and meiotic structure during mouse oocyte maturation
Source: Aging Cell. 2018 May 29;17(4):e12789. doi: 10.1111/acel.12789 (PMC6052465; doi:10.1111/acel.12789)
Supplement: Supplementary file 3 [file ACEL-17-na-s003.doc]

# Supplemental Table 1

# Primer sequences of genes for cDNA amplification

*Gene Primer sequence*

SIRT4

(1^st^ round) Forward primer: 5’- ACCCGACTGTTTAGCCGTTCA -3’

Reverse primer: 5’- ACTTCTGCCTGGTCCTTCTTCC -3’

SIRT4

(2^nd^ round) Forward primer: 5’-GGGGGCCGGCCCAGAATAAGAATGAGCGGATTG-3’

Reverse primer: 5’- GGGGGCGCGCCGCTTTGCCACCTCTAGGATT-3’

Primer sequences of genes for cDNA amplification

Gene Primer sequence

SIRT4-H158Y

Forward primer: 5’- GCTGACGGAGCTCTACGGATGCATGCACA -3’

Reverse primer: 5’ - TGTGCATGCATCCGTAGAGCTCCGTCAGC -3’

PDH-Ser293A

Forward Primer: 5’- CGCTACCATGGACACACCATGAGTGACCCTGGA– 3’

Reverse Primer: 5’- TCCAGGGTCACTCATGGTGTGTCCATGGTAGCG – 3’

Forward Primer: 5’ -CGCTACCATGGACACGCCATGAGTGACCCTGG– 3’

Reverse Primer: 5’- TCCAGGGTCACTCATGGCGTGTCCATGGTAGCG – 3’

Primer sequences for siRNAs

Gene Primer sequence

SIRT4 Forward Primer: 5’- UCA CUC AUU CAG GGC AUU UTT - 3’

Reverse Primer: 5’- AAA UGC CCU GAA UGA GUG ATT -3’

Control Forward Primer: 5’-UUCUCCGAACGUGUCACGUTT-3’

Reverse Primer: 5’-ACGUGACACGUUCGGAGAATT-3’
